# Lingonberries—General and Oral Effects on the Microbiome and Inflammation

**DOI:** 10.3390/nu13113738

**Published:** 2021-10-23

**Authors:** Pirjo Pärnänen, Hanna Lähteenmäki, Taina Tervahartiala, Ismo T. Räisänen, Timo Sorsa

**Affiliations:** 1 Department of Oral and Maxillofacial Diseases, Head and Neck Center, University of Helsinki and Helsinki University Hospital, P.O. Box 63 (Haartmaninkatu 8), FI-00014 Helsinki, Finland; hanna.b.lahteenmaki@helsinki.fi (H.L.); taina.tervahartiala@helsinki.fi (T.T.); ismo.raisanen@helsinki.fi (I.T.R.); timo.sorsa@helsinki.fi (T.S.); 2 Division of Periodontology, Department of Dental Medicine, Karolinska Institutet, 14104 Huddinge, Sweden

**Keywords:** Lingonberries, inflammation, oral microbiome, fermented lingonberry juice

## Abstract

Lingonberry (*Vaccinium vitis ideae* L.) is a low-bush wild plant found in the northern hemisphere. The berries are used in traditional medicine in Finland to treat oral yeast infections. General and oral effects of lingonberries on the microbiome and inflammation are reviewed. A brief introduction to oral microbiome symbiosis and dysbiosis, innate and adaptive immunity and inflammation are included, and special features in microbe/host interactions in the oral environment are considered. In vitro anticancer, antimicrobial, antioxidant, anti-inflammatory, and in vivo mouse and human studies are included, focusing on the symbiotic effect of lingonberries on oral and general health.

## 1. Symbiosis and Dysbiosis

**Figure 1:**
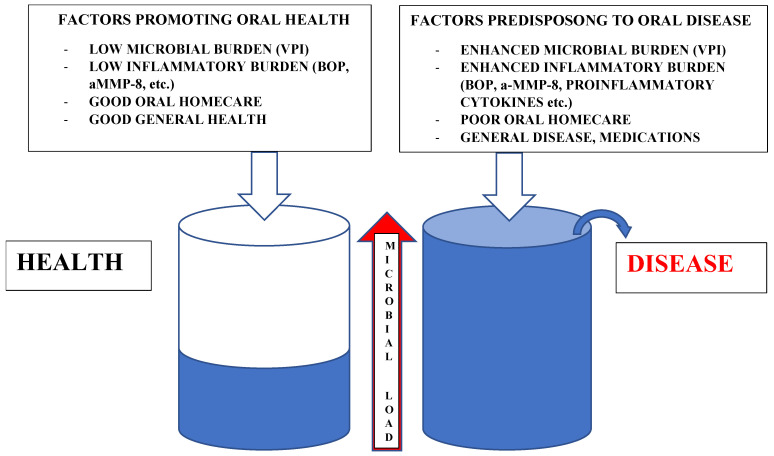
Illustration of some factors affecting healthy and diseased state in the oral environment. VPI (visible plaque index), BOP (bleeding on probing), aMMP-8 (active matrix metalloprotease-8).

Microbiome research acknowledges the concept of a symbiotic/dysbiotic microbiome in health and disease [1]. The normal microbiome competes constantly with pathogenic or opportunistic bacteria, yeasts and viruses for nutrients and attachment sites on host cells. The main predisposing factors for a dysbiotic microbiome, and consequently predisposition to a diseased state in the oral cavity, are summarized in Figure 1. The microbial load could be seen as an indicator of the risk in a patient of developing a diseased state; initially, high counts of opportunistic pathogens and predisposing factors, e.g., systemic disease, medications and low or immune incompetent saliva, do not tend to overcome the “reservoir” or tolerance of the individual to stay healthy. In the case of, e.g., host immunodeficiency, a dysbiotic microbiome with opportunistic pathogen overgrowth may occur, resulting in a diseased state. Broad-spectrum antibiotics may also disturb and interfere with the normal microbiome long after treatment has ended.

After birth, the oral normal microbiome starts to build up with primary microbial species colonizers, which are mainly *Viridans* group streptococci [2]: *Streptococcus salivarius* (*S. salivarius*) and *Streptococcus mitis* (*S. mitis*), and subsequently the secondary colonizers, which tend also to favor anaerobic growth conditions. The periodontopathogenic bacteria belonging to the red triad (*Porphyromonas gingivalis*, *Tannerella forsythia* and *Treponema denticola*) are found and occur due to advanced periodontal disease. Local tissue oxidative, nutrient or pH conditions may also often favor the coaggregation tendency of certain bacterial species in biofilm formation. Carious, periodontal, candidal or viral infections may follow if defense mechanisms of the host are insufficient and defective. Infections may develop from low grade inflammation to acute infections and even proceed to life-threatening sepsis.

## 2. Innate and Adaptive Immunity and Inflammation

The first few hours of interaction with antigenic microbes trigger the host innate immunity. Monocytes differentiate to macrophages and their receptors recognize microbial surface components: e.g., mannose, glucan, or lipopolysaccharide. Pathogen recognition triggers inflammatory responses, i.e., “circulus vitious inflammationis”. Macrophages engulf microbes fusing with lysosomes to lyse microbes and release toxic and oxidative products (e.g., hydrogen peroxide, superoxide anion, nitric oxide) and at the same time, proinflammatory prostaglandins, leukotrienes and platelet-activating factor (PAF), proinflammatory cytokines (e.g., TNF-α, IL-1, IL-6, IL-12) and chemokines (e.g., monocyte chemoattractant protein (MCP-1)) to attract neutrophils to extravasate and migrate, in order to degranulate to sites of inflammation. Inflammation-related molecules have been extensively reviewed by Joseph et al. [3]. Natural killer (NK) cells contain and kill intracellular pathogens until cytotoxic T-cells are generated. Activation of the complement system occurs, and recognition of microbes follows three different pathways (classical, manna-binding -lectin, alternative). This allows opsonization and destruction of microbes directly or indirectly by the phagocytes. Complement components C3a, C4a and C5a are proinflammatory peptide mediators.

Subsequently, adaptive immunity is triggered if the pathogens overcome innate immunity targeting to defend the host from potential pathogen invasion and actions. Dendritic cells engulf and degrade pathogens and maturate to antigen-presenting cells. Cytosolic pathogens are eliminated by cytotoxic T-cells recognized via co-receptor CD8 (viruses and cytosolic bacteria). CD4 T-cells recognize pathogens and their products derived from the cellular vesicular compartment. CD4 T-cells are specialized to activate other cells and fall into two classes: T-helper cells (TH1), inflammatory cells, activate macrophages; and TH2 cells activate B cells to start producing antibodies called immunoglobulins (Ig). Main classes of immunoglobulins are IgG, IgM, and secretory IgA in the oral cavity; the infection is thus eliminated from the extracellular space (adapted from Janeway et al. [4]).

## 3. Special Features in Microbe/Host Interactions in the Oral Environment

The oral microbiome consists of hundreds of yet not completely identified microbial species. A symbiotic oral mucosal microbiome is essential for the host’s healthy state. The epithelial surface, trying to obstruct invasion of the microbes, is at constant alert by bombardment of microbes and their virulence factors, such as adhesins, invasins and proteases, which may degrade oral epithelial junctional proteins, e.g., laminin-332, fibronectin, E-cadherin, and claudin [5,6,7,8,9,10], allowing further promotion of the microbial burden’s invasive process through the epithelium or degradation of complement proteins of innate immunity. Gingival junctional epithelium is especially vulnerable as its main structural basement membrane protein is laminin-332. 

Saliva and its antimicrobial substances, such as glycoproteins in mucus, lysozyme secreted by macrophages/neutrophils, β-defensins, lactoferrin, lactoperoxidase, statherin, histatins and secretory IgA [11] have an essential role in first line superficial defense. Matrix metalloprotease-8 (MMP-8), MMP-9, myeloperoxidase (MPO) and neutrophil (PMN) elastase have distinct roles in local and connective tissue collagen metabolism in the oral environment. The host exhibits housekeeping activity of MMP-1 and its inhibitors—tissue inhibitor of metalloproteases-1 and -2 (TIMP-1, TIMP-2)—as well as constant lysis and remodeling of collagenous structures. Oral microbes *Treponema denticola* [12,13] and *Candida glabrata* [14] have been shown to convert latent 75 kDa proMMP-8 to its lower molecular active and fragmented forms (aMMP -8), and elevated aMMP-8 activity in oral pathologies may especially cause undesirable and irreversible tissue damage. *Tannerella forsythia*, associated with severe periodontitis, is capable of degrading gelatin and type I collagen [15], and *Porphyromonas gingivalis* and *Treponema denticola* proteases, gingipain and dentisilin, respectively, as well as various other bacterial proteases activate human procollagenases [12,16]. Other non-matrix substrates of aMMP-8 are laminin-332 γ2- chain, insulin receptor, RANKL, TRAP, TREM-1: elevated concentrations of their proteolytic fragments may be found and detected during diseases and inflammations. Elevated levels of MMP-8 and MPO activity has been shown to correlate with poor prognosis of bacterial meningitis of childhood, pancreatitis, obesity, sepsis, and diabetes [17,18,19,20,21]. aMMP-8 may also be used as a biomarker of inflammation from oral rinse samples (PerioSafe^®^) or gingival crevicular fluid (GCF) in peri-implantitis (ImplantSafe^®^) [22]. If inflammation in periodontal tissues is prolonged, this low-grade inflammation, reflected by aMMP-8, may have also systemic effects on health, e.g., cardiovascular diseases, metabolic syndrome, Alzheimer’s disease, or autoimmune diseases [23,24].

Considering microbes and their infection/inflammation-inducing effects, proper management of oral health is crucial and exerts positive effects on general or systemic health. An acute infection’s spread may threaten general health. Low-grade inflammation induced and caused by persistent microbes is challenging because systemic antibiotics or local antiseptics may be used only for a limited time, and they abolish the normal microbiome alongside the pathogens. Natural products, and especially fermented plant foods, may eventually offer an alternative approach to novel anti-inflammatory and immunomodulatory pathways for controlling oral health [25].

## 4. Lingonberries

Lingonberry (*Vaccinium vitis ideae* L.) is a low-bush wild plant found in the northern hemisphere. The berries contain vitamins (A, B1, B2, B3, and C), potassium, calcium, magnesium, phosphorous and have a unique polyphenol composition [26], including flavonoids (anthocyanins, flavonols {e.g., quercetin}, flavanols {catechins])), phenolic acids, lignans, stilbenes (resveratrol), and phenolic polymers (e.g., proanthocyanidins)—of which anthocyanins, flavonols, and proanthocyanidines are the main constituents. Resveratrol may be extracted from berry peels and seeds. Lingonberries contain particularly high amounts of anthocyanins: cyanidin-3-galactoside (88%), cyanidin-3-arabinoside (10.6%) and cyanidin-3-glucoside (1.4%) [27]. Polyphenols from berries are bioavailable from diet [28] and they retain their biological activities in ileal samples [29]. Lingonberry bioactive molecules show anti-cancerous, antimicrobial, antioxidative and anti-inflammatory effects [30].

The following studies have used different fractions of lingonberries, depending on the extraction method used, and accordingly the antimicrobial effects seem to vary.

## 5. In Vitro Anticancer Studies

Hoornstra et al. [31] have demonstrated the inhibition of oral tongue squamous cell lines HSC-3 and SCC-25 carcinoma invasion and proliferation by fermented lingonberry juice similar that seen with curcumin. Lingonberries are known to exert ornithine decarboxylase inhibition, a key rate-limiting enzyme in polyamine synthesis, cell growth, DNA repair and carcinogenesis [32]. Lingonberries have additional antiproliferative effects against human breast, colon and cervical (HeLa) cancer growth [33,34,35].

## 6. In Vitro Antimicrobial Studies

There are limited amounts of studies on the effects of lingonberries against microbial growth; the most studied are intestinal pathogens and lactobacilli. Several antimicrobial mechanisms of polyphenols have been proposed [36,37,38]. Lingonberry polyphenols have also been proposed to act as antivirals [39], and in this regard, lingonberries have been shown to possess in vitro antiviral activity [40]. Other studies have also reported lingonberry’s antibacterial and antifungal activities: inhibition of growth of *Candida*, *S. mutans*, *Porphyromonas gingivalis*, *Fusobacterium nucleatum*, *S. aureus*, *Salmonella enterica* sv *Typhimurium*, *S. epidermidis*, *P. gingivalis*, *P. intermedia*, antiaggregation of *S. mutans* with *Fusobacterium nucleatum* or *Actinomyces naeslundii*, anti-adhesiveness of *Neisseria meningitidis* or oral streptococci in biofilm formation, and binding activity of *Streptococcus pneumoniae*, *Streptococcus agalactiae* and *Streptococcus suis* to berries and juices [36,41,42,43,44,45,46,47,48,49,50,51,52]. No effects of lingonberries on lactobacilli have been found in any of these studies.

## 7. In Vitro Antioxidant and Anti-Inflammatory Studies

Berries contain polyphenols which act as potent antioxidants and are neuroprotective, having beneficial effects on health [30,53]. Antioxidant activities of lingonberries have been assessed in vitro in several studies [41,54,55,56,57,58,59,60]. These studies report radical oxygen species scavenging by polyphenols and inhibition of oxidation of lipids and proteins.

**Figure 2:**
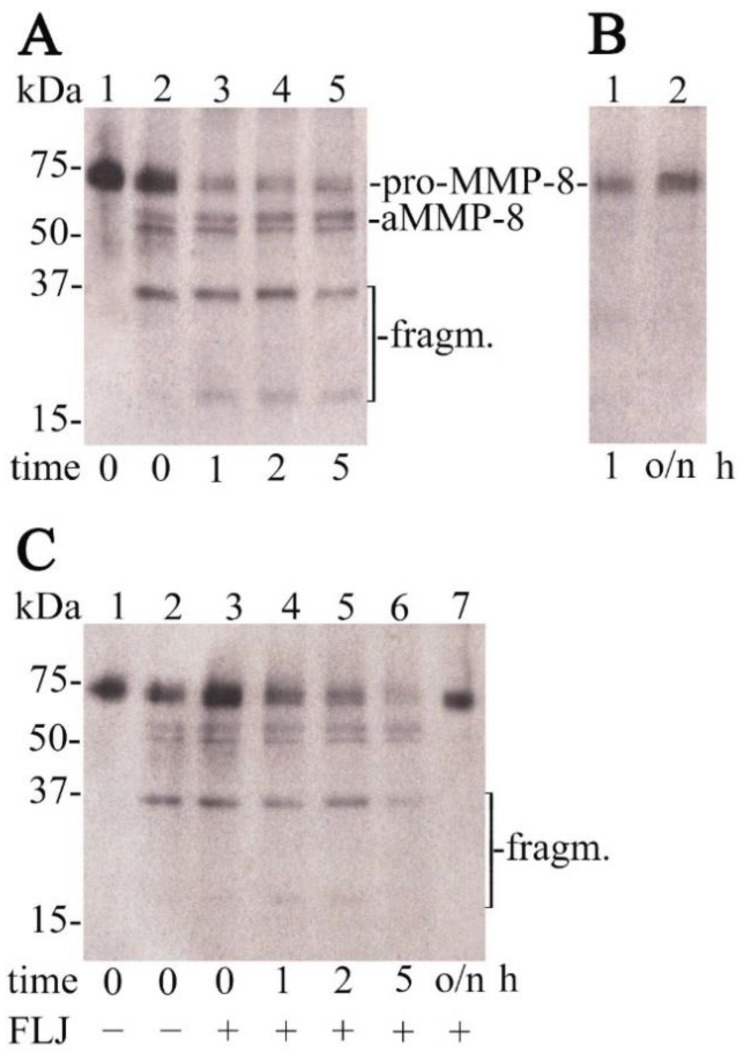
*Candida glabrata* T-1639 cell wall proteolytic fraction induced proMMP-8 activation, and inhibition of activation by fermented lingonberry juice (FLJ). (**A**). 1. proMMP-8 control, 2.–5. *Candida glabrata* T-1639 > 50 kDa fraction + proMMP-8, 0–5 h (incubated 37 °C). Fragmentation (activation) of proMMP-8 marked. (**B**). Lanes 1.–2. *Candida glabrata* T-1639 cell wall 10–50 kDa fraction + proMMP-8, 0 h-o/n (incubated 37 °C). No marked fragmentation of proMMP-8 is seen. (**C**). 1. proMMP-8 control, 2.–7. *Candida glabrata* T-1639 > 50 kDa fraction + proMMP-8 + FLJ, 0 h-o/n (incubated 37 °C). Inhibition of fragmentation of proMMP-8 can be seen. Molecular weight markers are indicated on the vertical axis. Performed according to Pärnänen et al. [14].

Several in vitro studies have also revealed anti-inflammatory mechanisms of lingonberries. Lingonberry cyanidin has been shown to inhibit in vitro p38 mitogen-activated protein kinase (MAPK) and c-Jun N-terminal kinase (JNK) phosphorylation, and reduce levels of protein kinase B (Akt) in UV-induced photoreceptor damage [61]. Lingonberry fruit extract has been shown in vitro to downregulate inflammatory mediators such as IL-6, TNF-α, IL-1β, MCP-1, COX-2 and iNOS in mouse adipocyte inflammation [62]. Lingonberry phenolic compounds suppress human THP-1 macrophage cells’ TNF-α and IL-6 production in vitro [41]. *Candida glabrata*’s cell-associated > 50 kDa proteolytic (gelatinolytic) fraction has been shown to activate human latent pro-MMP-8 [14], and this activation was time- and dose-dependently inhibited by FLJ (Figure 2). In vitro studies by Esposito et al. [63] revealed inhibition of reactive oxygen species (ROS) and NO production, and suppression of prostaglandin-endoperoxidase synthase 2 (COX-2) and inducible nitric oxid synthase (iNOS) expression in LPS-stimulated mouse macrophages. Additionally, human dermal fibroblast MMP2 and MCPI gene downregulation and upregulation of extracellular matrix protein-coding genes COLIA2 (pro-α2 chain of type I collagen), ITGB1 (integrin receptor subunit β1) and RHOA (guanosine triphosphate phosphatase) were detected, resulting in beneficial ECM remodeling, dermal wound healing and tissue repair based on earlier inflammatory resolution.

In vivo mouse studies have shown that lingonberry juice exerts anti-inflammatory and anti-atherothrombotic effects [64,65]. Lingonberries decrease glycaemia and hepatic triglyceride levels [66], reduce inflammation, high cholesterol, hyperglycaemia and obesity [67], and alter gut microbiota, improve metabolic/brain functions, and also reduce gut inflammatory properties [68] in mice fed a high-fat diet.

## 8. In Vivo Human Studies

In vivo human studies with lingonberries are scarce and few studies on consumption of mixtures of berries containing lingonberries have been conducted. One pilot intestinal study has been performed [29], which compared the bioavailability of polyphenols after in vitro digestion and following fecal fermentation of lingonberries in an in vivo digested lingonberry sample (ileal fluid sample of one male). The results showed that the (poly)phenol composition of lingonberry undergoes substantial but largely similar modifications in vivo and in vitro, retaining bioactivity in an anti-genotoxity model, in vitro samples also in a colorectal cancer model. It is proposed that the concentration rather than specific phenolic compounds is crucial to obtain effects.

Lingonberries or lingonberry nectar consumed with saccharose reduces postprandial glucose, insulin, and inflammation-related free fatty acid release responses, and lingonberry puree consumed with bread reduces insulin requirements, to maintain normal or slightly reduced postprandial glycaemia in healthy women [69,70].

**Figure 3:**
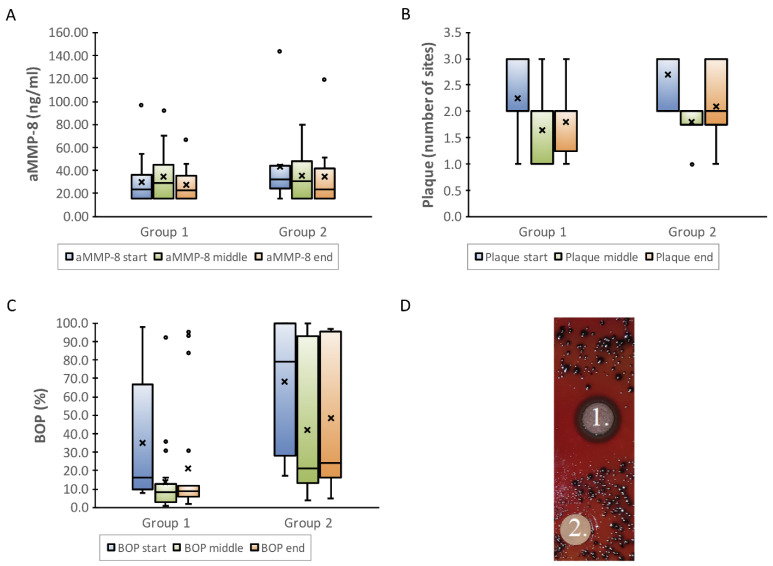
Inflammatory aMMP-8 and microbial visible plaque index (VPI), bleeding on probing (BOP) in vivo responses during fermented lingonberry juice (FLJ, Lingora^®^) mouthrinse trial. (**A**) aMMP-8, an oral fluid periodontal tissue destruction biomarker, represents and reflects inflammatory burden and periodontal disease activity, (**B**) VPI microbial burden and (**C**) BOP representing both inflammatory disease activity and microbial burden. Values of groups 1 and 2 at the start (0 d), middle (14 d, 7 d) and the end (28 d, 14 d). (**D**) Disc diffusion assay. Observed inhibition of *P. gingivalis* W50 growth with 100 µL FLJ (1.) compared to 10 µL 0.2% chlorhexidine (2.). Performed according to Pärnänen et al. [44]. The circles in the boxplots represent outlier values. “x” mean values.

Originally, concentrated unfermented lingonberry juice was tested among 60 patients (Finnish Utility Model nro 8195, 71). The randomly chosen adult patients used 10 mL of the juice as a mouthwash twice daily for ten days, after which the trial had to be discontinued because of an unexpected *Candida* count increases in vivo due to the high sugar content of the juice. In the next clinical studies, the sugar content of the juice was reduced, and lingonberries were shown to have in vivo antimicrobial and anti-inflammatory properties in the form of fermented lingonberry juice (FLJ; Lingora^®^, Vantaa, Finland) in clinical human oral studies [44,71] on a total of 40 adults. Initially, FLJ was lyophilized and compressed into tablets [71]. Ten individuals were divided into two groups according to pre-scanned *Candida* counts: those with higher *Candida* counts (≥ 10^4^ CFU/mL) took the lozenge three times daily, those with lower counts (< 10^4^ CFU/mL) took it twice daily for ten days. The results showed promising antimicrobial effects in vivo. In the next study, 30 patients were recruited randomly. The group was divided into two subgroups to test the suitable amount of FLJ and minimum time of the mouthwash period needed to gain antimicrobial and anti-inflammatory effects: 20 patients with clinically normal levels of oral homecare/plaque used 10 mL of FLJ twice daily for 2 weeks (group 1), and 10 patients with apparent difficulties in maintaining proper oral homecare/higher amounts of plaque used 20 mL twice daily for 7 days (group 2) as a mouthwash for 30 s. The results showed similar antimicrobial effects in both groups. Additionally, the on-line and real-time analysis and recording of clinical periodontal parameters, such as bleeding on probing (BOP, gingival inflammation index) and visible plaque index (VPI, microbial burden index), and the oral periodontal tissue destruction biomarker, aMMP-8 [72,73,74], reflected and indicated beneficial antimicrobial and anti-inflammatory changes (Figure 3).

Lingonberries are a promising natural approach with regards to their studied and recorded beneficial oral and general health effects via antioxidant, anti-inflammatory, anti-proteolytic, anticancerous and antimicrobial effects. Lingonberries, in specially formulated FLJ, have thus been designed for safe oral use as a mouthwash, and have thus been shown to exert anti-cancerous, antimicrobial, anti-proteolytic and anti-inflammatory properties in in vitro and in vivo clinical human studies. The antimicrobial effects of FLJ have been assessed against the fifteen most common oral microbial strains, including *Candida*, *Streptococci,* and key dysbiotic periodontopathogens. The species monitered in oral in vivo studies were selected to represent key microbes known to cause disease, e.g., *Candida* in candidosis and *S. mutans* in dental caries. Excessive amounts of *S. mutans* co-associate with the severity of periodontal disease in older patients [75]; the numbers of these species declined statistically significantly. In comparison, lactobacilli count results show that FLJ did not inhibit their growth. In fact, their relative proportion in patient samples increased after FLJ intervention, as expected, and the microbial effects of FLJ lasted even after the discontinuation of the mouthwash regimen. The overall effect on the microbiota assessed by these cultivations has interesting consequences regarding the composition of the oral microbiota: the patient’s own lactobacilli were allowed to flourish, cutting off living space and nutrients from potentially pathogenic species. Lactobacilli are indeed known to inhibit *S. mutans* and *Porphyromonas gingivalis* growth [76]. Microbes of the normal microbiome, such as *S. salivarius,* may also produce beneficial antimicrobial substances [2]. This kind of shift in the composition of the microbiota from dysbiosis (disease) towards symbiosis (health) could be beneficial and defensive to control oral health.

## 9. Conclusions

FLJ would have a beneficial effect, particularly in individuals who have predisposing factors (age, medication, dry mouth) or difficulties in maintaining sufficient oral homecare. As lingonberries contain considerable amounts of natural sugars, only the tailored fermented juice with the majority of sugars removed exerts this effect optimally. The antimicrobial and anti-inflammatory effects seem to continue after use, seem to have a prolonged treatment effect, and shift the oral microbiome to a more symbiotic direction. Double-blinded randomized trials are needed to study the effects on saliva parameters, dental caries, periodontal disease, and general health—since FLJ may and can be conveniently as well as safely swallowed—focusing on anti-inflammatory, anti-oxidative, and systemic effects mediated by, e.g., cytokine and reactive oxygen species levels.

## Data Availability

The data presented in this study are available on request from the corresponding author.
